# Psychiatric Co-Morbidities in Post-Traumatic Stress Disorder: Detailed Findings from the Adult Psychiatric Morbidity Survey in the English Population

**DOI:** 10.1007/s11126-020-09797-4

**Published:** 2020-07-23

**Authors:** T. Qassem, D. Aly-ElGabry, A. Alzarouni, K. Abdel-Aziz, Danilo Arnone

**Affiliations:** 1 Maudsley Health, Al-Amal Hospital, Dubai, United Arab Emirates; 2 Al-Amal Hospital, Ministry of Health and Prevention, Dubai, United Arab Emirates; 3 Okasha Institute of Psychiatry, Ain Shams University, Cairo, Egypt; 4 Mohammed Bin Rashid University of Medicine and Health Sciences (MBRU), Dubai, United Arab Emirates; 5 University of Nebraska, Lincoln, NE USA; 6 Department of Psychiatry and Behavioural Science, College of Medicine and Health Sciences, United Arab Emirates University, Al-Ain, United Arab Emirates; 7 Centre for Affective Disorders, Institute of Psychiatry, Psychology and Neuroscience, Psychological Medicine, King’s College London, London, UK

**Keywords:** Psychiatry, Post traumatic stress disorder, Comorbidities, Major depressive disorders, Survey

## Abstract

Post Traumatic Stress Disorder (PTSD) is a condition which causes great sufferance to the individuals affected. The occurrence of comorbidities in PTSD is a frequent event with a negative impact on outcome. This study investigated the frequency of PTSD in relation to comorbidities by analyzing the results of the 2007 ‘Adult Psychiatric Morbidity Survey’ in the English population, which included data on comorbidities. A population study conducted in the United Kingdom, this survey investigated the frequency of PTSD in the community and the relationship to comorbidities by adopting a random design to minimize selection bias, stratified by region and socioeconomic characteristics, and weighted according to design and non-response. The survey interviewed 7403 adults living in private households. Socio-demographic characteristics and psychiatric morbidity were systematically assessed. Results indicated that PTSD prevalence was 2.9%, with an excess in women (3.3%) compared to men (2.4%) as reported by the 2007 survey. Comorbidity was a very frequent occurrence in PTSD reaching 78.5% in affected cases. Major depression was the commonest condition and its frequency increased with symptoms severity up to 54%. Among anxiety disorders, social phobia was the most frequent, followed by generalized anxiety disorder, obsessive-compulsive disorder, agoraphobia and panic disorder. Substance use disorders were also common. The presence of psychotic symptoms was particularly significant with over 30% prevalence in PTSD. These results indicate that attention needs to be devoted to the presence of comorbidities. In view of the impact of comorbidities on PTSD severity, chronicity and functional impairment, early detection and treatment are likely to improve outcome.

**Electronic supplementary material:**

The online version of this article (10.1007/s11126-020-09797-4) contains supplementary material, which is available to authorized users.

## Introduction

The co-occurrence of psychiatric morbidity is significant in PTSD [1–5] with concerning estimates for major depression reaching 48% of PTSD cases [6], often coexisting with suicidal ideation [7], the frequent presence of symptoms of generalized anxiety disorder [8] and of alcohol dependence with increased levels of aggression and impulsivity [6, 9–12]. The association between PTSD and psychosis is also recognized as contributing to the burden and quality of life of patients with schizophrenia [13] and in some studies is related to negative symptoms [14]. Despite evidence of a significant negative interaction between comorbidities and the outcome of PTSD [15], there is limited information available in the literature from sufficiently powered studies to appraise the frequencies of comorbidities at population level [16] particularly from European samples. This is relevant because differences in PTSD prevalence shown globally [1, 16] might also reflect on comorbidities [17]. More accurate information, based on data from local samples, is essential for devising more precise care models and tailored services for more complex forms of PTSD [18]. This work reports PTSD comorbidity data from the 2007 ‘Adult Psychiatric Morbidity Survey’ in the English population and provides a detailed description of the concomitant psychopathology when PTSD criteria are met.

## Methods

### Patient Population

Strengthening the Reporting of Observational Studies in Epidemiology (STROBE) Statement guidelines for reporting observational studies were followed [19]. This study presents data from the 2007 adult psychiatric morbidity survey [20] which included individuals aged 16–64 living in the UK. The survey interviewed 7403 individuals, a sample size sufficient to detect less common psychiatric disorders (0.5–1.0%) by taking into account age, sex and UK region [20].

### Assessment and Data Collection

Adults living in private households were sampled using a population-based multi-phase probability sampling (Small User Postcode Address File approach). Well trained survey interviewers identified private households after one person in the household had been selected at random to minimize selection bias (Kish grid method) [21]. Furthermore, the sample was stratified by region and socioeconomic characteristics and weighted for survey design and non-response [22]. Ethnic categories were classified as ‘white’ which included all white participants independent of their country of origin, ‘black’ comprising Caribbean, black African, black other, and mixed white/black, and a residual ‘Other’ group. Social class was classified according to the UK Registrar General’s classification, and divided into three groups: social classes I and II, social class III, and social classes IV and V (this last group also included members of the armed forces).

During phase 1 of the survey, socio-demographic characteristics were collected and suitability for phase 2 was established with a detailed questionnaire. In phase 2 interviews were carried out by clinically trained researchers. The Revised Clinical Interview Schedule (CIS-R) [23] was used to establish the presentation of prevalent current mental disorders. A diagnostic algorithm was utilized to establish ICD-10 diagnoses including depressive episodes, generalized anxiety disorder, mixed anxiety and depressive disorder, panic disorder, phobias and obsessive–compulsive disorder [21]. The possible presence of psychotic symptoms was assessed during phase 1 by using the Psychosis Screening Questionnaire (PSQ) [24] and subsequently confirmed at phase 2 in a formal assessment based on set criteria (see Supplementary material). The experience of a traumatic events was systematically assessed with the non-patient version of the Structured Clinical Interview for DSM-IV (SCID) [25]. If any event occurred from the age of 16 onwards, PTSD experiences were explored by using the Trauma Screening Questionnaire (TSQ) [26]. The TSQ is a valid instrument to screen for PTSD symptoms in primary care according to the National Institute of Clinical Excellence aligned with DSM IV criteria [21] with a sensitivity of 84%, a specificity of 95% and a positive predictive value for PTSD vs. other forms of trauma of 90% [21, 26]. The Alcohol Use Disorders Identification Test (AUDIT) [27], the community version of the Severity of Alcohol Dependence Questionnaire (SADQ-C) [28] and the Comprehensive Addiction Severity, based on the Diagnostic Interview Schedule [29] were used to assess and identify alcohol misuse over the preceding 6 months and drug use over the past year.

### Statistical Analysis

Data from each survey were weighted to allow for design and response rates [21, 30, 31] and analyzed using the Data Analysis & Statistical Software (STATA 15; StataCorp. 2017. Stata Statistical Software: Release 15. College Station, TX: StataCorp LLC.). Binary logistic regression analyses were used to calculate the odds ratios (ORs) and 95% confidence intervals (CIs).

### Patient and Public Involvement

Patients or public were not involved in the design of this study.

### Compliance with Ethical Standards

The Royal Free Medical School Research Ethics Committee (London, UK) granted Ethical approval for the Adult Psychiatric Morbidity Survey 2007 (Ref.: 06/Q0501/71).

## Results

### Characteristics of the Sample and Prevalence of PTSD

Overall 7325 participants were eligible, 3163 men (43.2%) and 4162 women (56.8%). Two hundred and thirteen individuals (2.9%), 75 men (2.4%) and 138 women (3.3%) met the criteria for a probable PTSD diagnosis (mean age 42.9 years, SD = 15.0). White middle class participants were more represented in the groups (see Table 1 for details).

**Table 1:** Characteristics of the sample

	Probable PTSD	
	Not Present (*n* = 7112)	Present (*n* = 213)
Males %	97.4%	2.6%
Females %	96.8%	3.2%
Age, mean (SD)	51.3 (18.5)	42.9 (15.0)
Upper class N (%)	2570	64 (2.2)
Middle class N (%)	3724	124 (3.1)
Lower class N (%)	354	6 (2.0)
White N (%)	6575	191 (2.8)
Black N (%)	180	8 (6.1)
Other ethnicities N (%)	337	12 (2.2)

### Co-Morbid Psychiatric Diagnoses

**Fig. 1:**
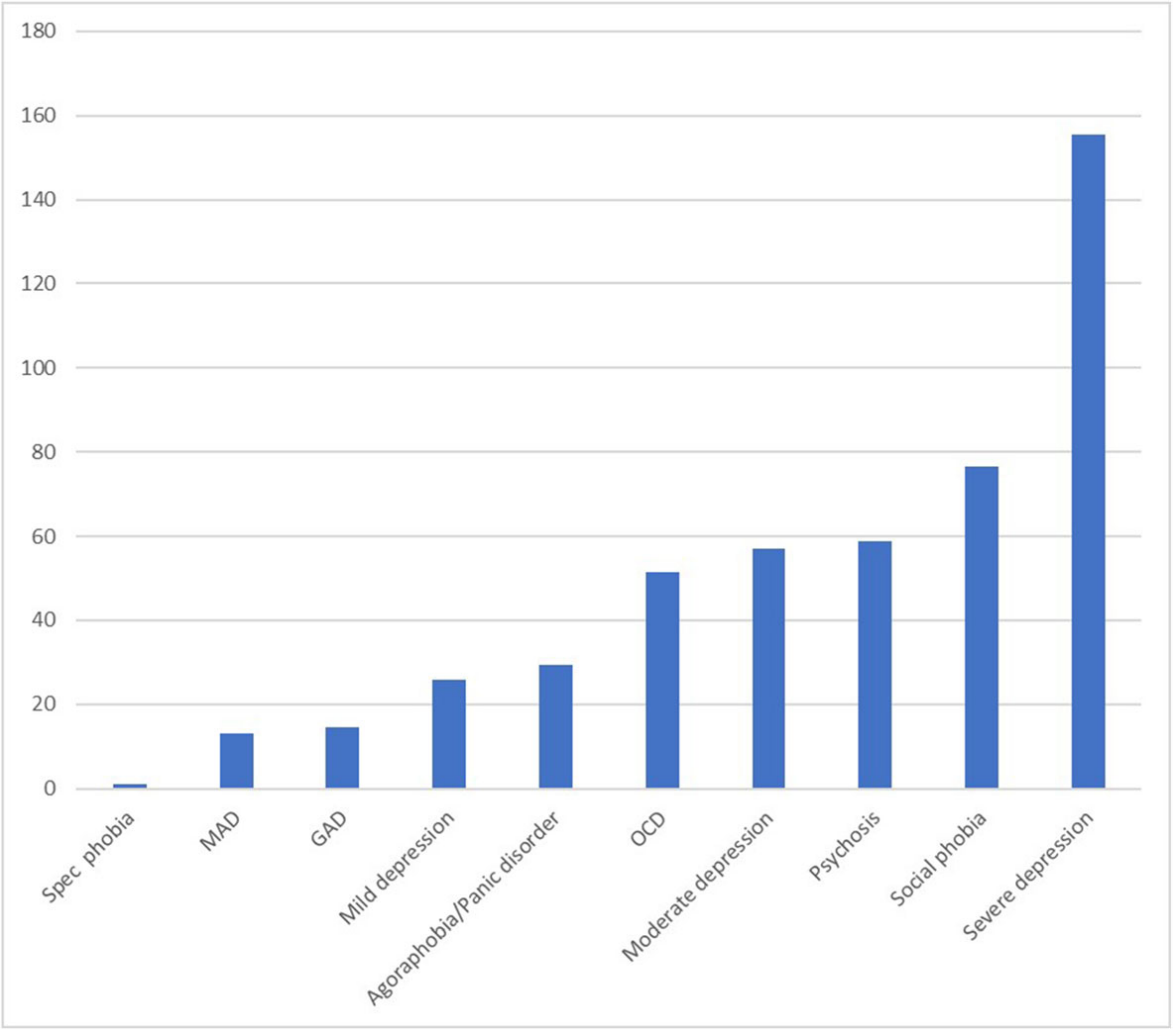
Strength of association between PTSD diagnosis and other comorbidities as indicated by their odds ratio (OR)

The co-existence of comorbid psychiatric diagnoses in the individuals with probable PTSD was 78.5% compared to 14.5% in those who did meet PTSD criteria. Table 2 and Fig. 1 show the distribution and frequency of comorbid conditions. The odds ratio indicated a significant association with most conditions except for specific isolated phobias. Depressive disorders were the most represented with increasing severity (16.2%–53.6%), followed by social phobia (36.3%), psychotic symptoms (30.4%), obsessive-compulsive disorder (27.7%), agoraphobia/panic disorders (17.9%), generalized anxiety disorder (9.8%), alcohol misuse and dependency (9.5%), and use of substances (12.6%).

**Table 2:** Psychiatric diagnoses predicting the occurrence of probable Post Traumatic Stress Disorder (PTSD); OR = Odds Ratio

Other psychiatric comorbidities	Probable PTSD		Odds Ratio	z	*P* value	Confidence Interval
	Not Present (*N* = 7112) %	Present (N = 213) %				
No other mental disorder *N* = 6066	99.3	0.7				
Mixed Anxiety and Depression *N* = 665	91	9	13.3	11.0	<0.001	8.4–21.1
Mild depression *N* = 55	83.8	16.2	25.9	7.1	<0.001	10.6–63.8
Moderate depression *N* = 122	70.1	29.9	57.2	14.0	<0.001	32.5–100.6
Severe depression N = 7	46.4	53.6	155.4	6.0	<0.001	30.0–804.2
Agoraphobia/Panic disorder *N* = 84	82.1	17.9	29.3	11.0	<0.001	14.0–61.1
Obsessive Compulsive Disorder *N* = 34	72.3	27.7	51.5	7.8	<0.001	19.1–138.9
Social phobia *N* = 37	63.7	36.3	76.6	10.11	<0.001	33.1–177.8
Generalized Anxiety Disorder *N* = 205	90.2	9.8	14.6	8.6	<0.001	7.9–27.0
Specific Isolated Phobias *N* = 13	100	0.0	1.0	NA	NA	NA
Psychosis N = 37	69.6	30.4	58.7	9.7	<0.001	25.8–133.7
Alcohol misuse/dependence N = 20	90.5	9.5	4.1	6.6	<0.001	2.7–6.4
Substance use disorders *N* = 27	87.4	12.6	5.5	6.5	<0.001	3.3–9.3

## Discussion

We set out to investigate the frequencies of PTSD and explore its comorbidities. As reported by the 2007 ‘Adult Psychiatric Morbidity Survey’ in the English population the prevalence of PTSD equaled 2.9%, with an excess in women (3.3%) compared to men (2.4%). White middle class participants were more represented in the groups. In agreement with previous research, we found that comorbidity is a very frequent occurrence in PTSD reaching 78.5% in this survey. Major depression was the commonest condition and its frequency raised with increasing severity, up to 54% in case of severe symptoms. We also found that among anxiety disorders, social phobia had the highest comorbidity with PTSD compared to generalized anxiety disorder, obsessive-compulsive disorder, agoraphobia and panic disorder. We also found significant levels of substance use disorders. The presence of psychotic symptoms was particularly significant with over 30% prevalence in PTSD. This is of interest given the small number of large surveys, which systematically assessed the presence of psychotic symptoms in individuals with PTSD. It is possible that this finding is related to the design of this study powered to detect less common psychopathology combined with the psychometric tools used.

It is not possible for this work to explain the high rate of comorbidities and provide potential etiological explanations. It is plausible that depressive disorders as well as anxiety symptoms can be a consequence of PTSD as well as an independent risk factor for PTSD following trauma exposure. Both conditions could also be a vulnerability factor for developing PTSD once exposure to traumatic events occurs. The relationship between PTSD and the use of alcohol and substances could be understood as a coping strategy to attenuate disturbing PTSD symptoms according to the self-medication hypothesis [32]. Regarding psychotic symptoms, there is a complex relationship with PTSD. Firstly, some of the psychotic symptoms can occur in case of severe PTSD. For example, thoughts which could be described as persecutory, the occurrence of abnormal multimodal perceptions [33] and the phenomenon of depersonalization/derealisation have been described in PTSD [34, 35]. This has led to conceiving a subtype of PTSD with psychotic symptoms as a way of explaining psychopathology. Other explanations are however possible, including that trauma could trigger PTSD as well schizophrenia and there is evidence that PTSD could be a prodromal manifestation of Schizophrenia [35, 36]. It is not to underestimate though that psychotic symptoms could be related to misusing substances, and associated with intoxication, be the result of withdrawal symptoms or complications related to chronic use (e.g. alcohol hallucinosis) [32].

It is essential to reflect on the possible overlap of the diagnostic criteria between PTSD and other mental disorders. The significantly high rates of comorbidity detected may be a reflection of the limitations of the diagnostic criteria used [32]. This is particularly relevant when it is not possible to establish a temporal relationship with the onset of the symptoms. Kessler and colleagues in their landmark survey also reported high rates of comorbidities in their study and similarly to this study, could not establish the primacy of PTSD [16]. It is possible that the high degree of symptoms overlap across syndromes contributes to under diagnose PTSD, particularly when detailed histories of traumatic events are not fully elicited. It is therefore important in routine clinical practice to specifically ascertain or exclude the presence and significance of traumatic events to avoid underestimating or overestimating PTSD in relation to other disorders. It may increase discriminative power to focus on the more distinctive symptoms of PTSD, particularly those not overlapping with other psychiatric conditions e.g. history of trauma, the presence of flashback and the individual response to traumatic events [16]. Algorithms that implement numerical weights for counting repeated items in assessment inventories could help reduce diagnostic overlap [37]. Another way might be to amend the diagnostic overlap by re-examining the constructs that define PTSD [38]. Rosen and Lilienfeld focused on the absence of valid empirical support for diagnostic criteria for the disorder [37]. This observation would be consistent with some studies that have referred to the fact that the onset of the classic form of trauma as a main diagnostic component is neither sufficient nor necessary for the onset of PTSD [39, 40]. Furthermore, some of the current literature alludes to the diagnostic boundaries of PTSD not being as specific as it is purported to be [38, 39, 41]. For example, many non-traumatic stressors (e.g. divorce of one’s parents, relationship problems) could lead to a higher number of PTSD symptoms when compared to the typical trauma described by diagnostic manuals, although extending the diagnostic boundaries of PTSD by lowering the threshold for the definition of trauma may not result in better precision [40, 42].

### Limitations

Large surveys such as this one offer the great opportunity to appraise conditions such as PTSD at population level by estimating disease prevalence over a time period. The main limitation of this approach is that cross sectional data do not allow for a prognostic evaluation and cannot establish primacy. This information can only be obtained from longitudinal data. Nevertheless, it is known that meeting criteria for more than one psychiatric disorder increases severity, prolongs illness, service utilisation, and negatively affects functionality [3, 43]. In this context, early intervention and recognition of comorbidities is likely to have a significant public health impact to reduce morbidity and improve outcome.

## Conclusion

In conclusion, this large population based survey reports a frequency of PTSD around 3% in the English population with an excess in women compared to men. Comorbidity in PTSD is highly prevalent and major depression is the most frequent among the detected mental health conditions. Psychotic symptoms were surprisingly common in PTSD. Exploration of comorbidities warrants further investigation because early recognition might prove beneficial to reduce morbidity, chronicity and loss of function. Addressing comorbid conditions in PTSD might also provide an insight into optimizing diagnostic criteria in view of the diagnostic overlap with some of the frequently occurring syndromes. Furthermore, a better understanding of the role of co-morbidities in PTSD could provide a rationale for subtyping PTSD to optimize treatment outcomes. Some attention could also be devoted to understanding some of the potential moderating factors including those of biological nature e.g. the relationship between immunology and the stress response [44], which might explain the overlap between PTSD and some of the comorbid conditions. Further studies with a longitudinal design assessing the primacy of one disorder over the other could help disentangle temporal associations.

## Electronic supplementary material

ESM 1 (DOCX 15.2 kb)
