# Supplementary material for: Psychiatric Co-Morbidities in Post-Traumatic Stress Disorder: Detailed Findings from the Adult Psychiatric Morbidity Survey in the English Population
Source: Psychiatr Q. 2020 Jul 23;92(1):321–30. doi: 10.1007/s11126-020-09797-4 (PMC7904722; doi:10.1007/s11126-020-09797-4)
Supplement: Supplementary file 1 — (DOCX 15.2 kb) [file 11126_2020_9797_MOESM1_ESM.docx]

**Supplementary information**

1. ***Criteria for confirming psychosis***

• Currently using any form of anti-psychotic medication.

• An in-patient for a mental or emotional problem in the past three months, or admission to a mental health unit at any time.

• An affirmative “yes” response to question 5a in the PSQ. This response is tied to auditory hallucinations.

• If the diagnosis of psychosis or any symptoms related to the disorder were self-reported by the participant.

To facilitate the process of coding during analysis, participating subjects who did not meet any of the above-mentioned criteria were not considered to have psychosis.

1. ***Post-Traumatic Stress Disorder Screening questions***

‘Have you ever being around or experiencing a natural disaster, a serious automobile accident, major sexual abuse, witnessing someone killed or seriously injured, having a loved one pass away by murder or suicide, or any other experience that either places the participant or someone close to him at a grave risk of harm or death.’

Participants who responded ‘yes’ to the above questions were asked about the time of the traumatic event.

1. ***Trauma Screening Questionnaire***

This is 10-item questionnaire that requires participants to provide either a "yes" or "no" response. The first five items tap the reliving of the traumatic event, while the second five items assess the experience of arousal after the traumatic event. In this study, all 10 items were used in the analysis. Some of presented statements included ‘upsetting thoughts or memories about the event that have come into your mind against your will’ and ‘irritability or outbursts of anger’. Respondents were probed to choose either ‘yes’ or ‘no’ to the statements. One point was given for a ‘yes’ response to each item, and a total of 56 points from the 10 items would prompt a positive screen for PTSD.

1. ***Use of illegal substances***

Participants were asked five questions that were meant to measure drug dependence. These questions covered level of use, sense of dependency, inability to abstain or continuous instances of relapse, increased level of tolerance and apparent withdrawal symptoms. Drug Use Disorder was established by having the participant endorse any of the items above as present within the past year.
